# Dilute Semiflexible Polymers with Attraction: Collapse, Folding and Aggregation

**DOI:** 10.3390/polym8090333

**Published:** 2016-09-06

**Authors:** Johannes Zierenberg, Martin Marenz, Wolfhard Janke

**Affiliations:** Institut für Theoretische Physik, Universität Leipzig, Postfach 100 920, Leipzig D-04009, Germany; johannes.zierenberg@itp.uni-leipzig.de (J.Z.); martin.marenz@itp.uni-leipzig.de (M.M.)

**Keywords:** semiflexible polymers, structural phases, collapse, aggregation, knots

## Abstract

We review the current state on the thermodynamic behavior and structural phases of self- and mutually-attractive dilute semiflexible polymers that undergo temperature-driven transitions. In extreme dilution, polymers may be considered isolated, and this single polymer undergoes a collapse or folding transition depending on the internal structure. This may go as far as to stable knot phases. Adding polymers results in aggregation, where structural motifs again depend on the internal structure. We discuss in detail the effect of semiflexibility on the collapse and aggregation transition and provide perspectives for interesting future investigations.

## 1. Introduction

Understanding the basics of polymer chemistry and physics has been a subject of research for many decades. The fundamental macroscopic equilibrium properties are well described both from a static and dynamic point of view [1,2,3,4]. The understanding of microscopic processes and the involved transitions was facilitated by employing computer simulations, which have been drastically improved over the last few decades. This approach is based on a (microscopic) statistical mechanics formulation of phase space with a proper Hamiltonian that incorporates all relevant interactions. Of course, this step may involve simplifications, such as implicit solvents or coarse-graining, to focus on the key processes of interest. The main computational approaches may be grouped into molecular dynamics (MD) simulations [5,6,7] and Monte Carlo (MC) methods [6,8,9,10,11]. MD is based on numerical integration of Newton’s equations of motion and delivers information on the thermodynamics, structure and dynamics from trajectories in phase space. In contrast, MC is based on stochastic sampling of phase space in the ensemble formulation of statistical physics. Direct dynamical information is traded with the possibility to define suitable move sets between microstates and the flexibility to devise generalized ensembles that are especially tailored to the problem at hand. This greatly improves the accuracy, often by many orders of magnitudes. We focus in this work on the developments and application of the latter approach with special emphasis on the thermodynamic and structural properties of polymeric systems.

We confine our discussion to linear coarse-grained polymers at low density. This excludes topics, such as polymer melts [12], polymer networks [13] and polymer nanocomposites [14]. We assume implicit solvents and incorporate excluded volume, self- and mutual-attraction, as well as semiflexibility. This approach connects chemical or synthetic polymers, which can be rather flexible, and biopolymers, which are commonly rather stiff.

## 2. Off-Lattice Polymer Models with Attractive Interaction

There is of course a whole zoo of models available for the study of semiflexible polymers. These range from lattice models, such as the (interactive) self-avoiding walk [15] or the bond-fluctuation model [16,17], over off-lattice models to analytic formulations, such as the (discrete) worm-like chain [18]. Furthermore, the extension with self- and mutual-interactions is possible in all formulations. We focus in this work on coarse-grained off-lattice polymer models, consisting of linearly-connected beads. Self-avoidance and short-range attraction are modeled by a Lennard–Jones, Morse or related interaction potential, e.g., of the form:

(1) $$V_{\mathrm{LJ}}\left(r\right)=4\epsilon \left[{\left(\sigma /r\right)}^{12}-{\left(\sigma /r\right)}^{6}\right].$$

This commonly sets the energy and length scale. Semiflexibility is usually introduced by a worm-like chain-motivated bending energy penalty of the form:

(2) $$V_{\mathrm{Bend}}\left(\theta \right)=\kappa \left(1-\cos\theta \right),$$

where *θ* is the angle between two successive bonds. A last detail involves the rigidity of the bonds by which the beads are connected, which may be considered either as sticks or springs. The latter may be approximated by harmonic or anharmonic springs, e.g., with the finitely-extensible nonlinear elastic (FENE) potential:

(3) $$V_{\mathrm{FENE}}\left(r\right)=-\frac{K}{2}R^{2}\ln\left(1-{\left[\left(r-r_{0}\right)/R\right]}^{2}\right).$$

The explicit results we present below in Section 4 and Section 5 are mainly based on two polymer models, namely a bead-stick and a bead-spring model. Our bead-stick model has rigid bonds of length $r_{b}=1$, and all beads interact with the Lennard–Jones potential, where σ=1 and ϵ=1. In our bead-spring model, the bonds are modeled with the FENE potential, and we consider Lennard–Jones interactions only between non-bonded monomers, where ϵ=1, $r_{0}=0.7$, $\sigma =2^{-1/6}r_{0}$, R=0.3, K=40, following the convention of Milchev, Binder and co-workers [19,20]. This choice introduces a substantial difference between bonded and non-bonded interactions. Other approaches model bonds by combining FENE and Lennard–Jones interactions (e.g., the Kremer–Grest model [21]). This has been shown to allow low-temperature Lennard–Jones crystal behavior in polymers [22]. For numerical reasons and in order to be consistent with previous literature, the Lennard–Jones potential is cutoff and shifted at $r_{c}=2.5\sigma$. Semiflexibility is adjusted in both cases by varying *κ* in $V_{\mathrm{Bend}}\left(\theta \right)$.

## 3. Monte Carlo Simulation and Analysis Methods

The study of structural phases in polymeric systems generally involves complex, possibly entangled, structured states. When interested in static properties, Markov chain Monte Carlo methods are a perfect tool to sample the conformational phase space. A well-known and often employed realization is the Metropolis algorithm [23] with advanced extensions, such as parallel tempering [24,25,26,27]. Not uncommonly, the involved transitions may be classified as first-order like, calling for advanced simulation techniques, such as generalized-ensemble methods. These may be roughly classified in flat-histogram methods and locally-confined-histogram methods. Especially in the dilute regime, the enhanced conformational entropy causes computational effort. For numerical purposes, one often reduces the consideration to the conformational phase space, i.e., the potential energy $E_{p}$ instead of total energy *E*. This is legitimate for systems where the momentum part may be integrated explicitly. There also exists a broad range of modified or generalized dynamic approaches, such as molecular dynamics in the multicanonical ensemble [28], metadynamics [29] and statistical temperature molecular dynamics [30]. These rely on molecular dynamics and will not be discussed in the present scope; instead, we refer to [31] for a recent comparison.

A crucial aspect that we merely want to mention is the choice of suitable Monte Carlo updates for computer simulations of polymers. This makes the difference between a well-equilibrated successful simulation and one that does not yield sensible results. Suitable updates are extremely model dependent and may include local bead displacement, long-range polymer displacement (important for dilute aggregation), pivot rotations [32,33] or local-bond rotations [34] and double-bridging moves [35]. A cleverly-designed move set may lead to substantial advances in numerical performance.

### 3.1. Generalized-Ensemble Methods: Flat Histogram

Generalized-ensemble (GE) methods have a long history, with early contributions, such as umbrella sampling [36], and later, flat-histogram formulations, such as multicanonical [37,38,39,40], Wang–Landau [41,42], statistical-temperature Monte Carlo [30], stochastic approximation Monte Carlo (SAMC) [43,44] and 1/t [45] sampling. Recall that in the canonical ensemble, the overall weight of a specific potential energy is decomposed into the amount of available conformational phase space, i.e., the conformational density of states $\Omega (E_{p})$, and the Boltzmann weight. This is expressed in the partition function:

(4) $$Z_{\mathrm{can}}=\int dE_{p}\Omega \left(E_{p}\right)e^{-\beta E_{p}},$$

where $\beta =1/k_{B}T$ is the inverse temperature, with $k_{B}$ the Boltzmann constant. The basic idea of generalized-ensemble methods is to modify the Boltzmann weight, such that the overall weight of specific energies is enhanced (e.g., for transition states) or decreased (e.g., for unimportant states). We may directly introduce a generalized weight function $W_{\mathrm{GE}}\left(E_{p}\right)$, which leads to a generalized partition function:

(5) $$Z_{\mathrm{GE}}=\int dE_{p}\Omega \left(E_{p}\right)W_{\mathrm{GE}}\left(E_{p}\right).$$

A common approach in modern flat-histogram applications is now to approximate $\Omega (E_{p})$ iteratively. If we choose $W_{\mathrm{GE}}\left(E_{p}\right)\approx \Omega {\left(E_{p}\right)}^{-1}$, this allows the sampling of a full range of potential energies with roughly equal weight in a final production run. For a recent review on flat-histogram methods in computer simulations of macromolecules, see [46].

Due to the current development of computational resources, it is advisable to consider parallel implementations of generalized-ensemble methods. The multicanonical method may be easily extended to parallel architectures and profits from the contributions of independent Markov chains to the estimate of a mutual probability distribution [47]. The parallelization becomes more cumbersome for Wang–Landau simulations, but introducing a clever energy-window distribution of walkers ensures good performance [48]. This is similarly possible for the 1/t algorithm [49].

### 3.2. Generalized-Ensemble Methods: Locally-Confined Histograms

Another approach could be summarized as locally-confined histogram methods, where the choice of suitable parameters locally confines the probability distribution to a specific potential-energy range. A physically-motivated example is microcanonical sampling [50,51], where the conformational phase space (potential energy) is extended by the momentum phase space (kinetic energy). Explicit integration of the momentum part yields:

(6) $$W_{\mathrm{GE}}=W_{\mathrm{NVE}}={\left(E-E_{p}\right)}^{(N_{\mathrm{dof}}-2)/2},$$

where $N_{\mathrm{dof}}$ is the total number of degrees of freedom in momentum space (e.g., for *N* independent particles $N_{\mathrm{dof}}=3N$). In this scenario, the total energy is kept constant, and the kinetic energy acts as a reservoir, from which energy may be added to (or removed from) the potential energy. This is in strong contrast to the heat bath of the canonical ensemble. Conceptually similar formulations include the Gaussian modified ensemble [52] and the generalized replica-exchange method [53]. In all of these cases, one typically obtains Gaussian-like potential-energy distributions for different physical or unphysical control parameters. As a benefit for first-order-like transitions, the transition states in this generalized ensemble are less suppressed if present at all. A detailed discussion may be found in [54].

These approaches may be trivially combined with a replica-exchange scheme to sample multiple control parameters in parallel, but with overlapping histograms. Moreover, a generalized weighted histogram analysis method (WHAM) [51,55,56,57] allows an estimation of the density of states and, thus, opens a route to reweight back into the canonical ensemble analogous to the discussion in the following subsection.

### 3.3. Reweighting from Generalized Ensembles

If the final data stem from an equilibrated Markov chain Monte Carlo simulation, i.e., with a fixed set of weights $W(E_{p})$, then it is possible to reweight the data to obtain estimates of canonical expectation values. A necessary condition is that the relevant ranges of the desired canonical potential-energy probability distribution are covered by the sampled histogram. The canonical expectation value is obtained from a generalized ensemble as:

(7) $${\langle O\rangle }_{\beta }=\frac{{\langle Oe^{-\beta E_{p}}/W_{\mathrm{GE}}\left(E_{p}\right)\rangle }_{\mathrm{GE}}}{{\langle e^{-\beta E_{p}}/W_{\mathrm{GE}}\left(E_{p}\right)\rangle }_{\mathrm{GE}}}.$$

In the following, we set $k_{B}=1$, which together with ϵ=1 in Equation (1) leads to a dimensionless temperature. It is common to discretize $W_{\mathrm{GE}}\left(E_{p}\right)$ despite having continuous energy domains. While this may introduce sampling problems if the energy bins are too large, it does not introduce systematic errors in the reweighting of the time series. Due to the fixed weights, the explicit (discrete) simulation weight can be simply divided out, and the desired (continuous) weight is multiplied to the observable for each measurement in the dataset. Error bars may be estimated in the same way by applying the jackknife or bootstrap method [58,59].

### 3.4. Canonical and Microcanonical Analysis

For semiflexible polymers, there is not necessarily a clear path towards a thermodynamic limit because of the finite nature of the system of interest. Therefore, a classification of transitions for finite systems becomes relevant. In most cases, a proper combination of canonical and microcanonical analysis yields the most concise picture.

The signatures of a structural phase transition are usually well identified as peaks in the thermal derivatives of canonical observables, such as the specific heat $C_{V}=\left(d\langle E\rangle /dT\right)/V$. The thermal derivative is obtained as $d\langle O\rangle /dT=k_{B}\beta^{2}\left(\langle OE\rangle -\langle O\rangle \langle E\rangle \right)$. However, sometimes, signals are not as clear. The collapse transition in off-lattice polymers, for example, may only show a shoulder in the specific heat, but shows a clear signal in the thermal derivative of the squared radius of gyration $R_{\mathrm{gyr}}^{2}=\sum_{i=1}^{N}{\left(r_{i}-r_{\mathrm{cm}}\right)}^{2}/N$ or the end-to-end distance $R_{\mathrm{ee}}=\left|r_{1}-r_{N}\right|$, where $r_{i}$ is the position vector of the *i*-th monomer and $r_{\mathrm{cm}}$ is the center-of-mass vector. In other cases, it is advisable to introduce explicit order parameters that capture the expected changes that occur during the transition, e.g., the phase-separation parameter $\Gamma^{2}=\frac{1}{2M^{2}}\sum_{i,j}{\left(r_{\mathrm{cm}}^{i}-r_{\mathrm{cm}}^{j}\right)}^{2}$ for aggregation, where the superscript refers to one of the *M* polymers. If multiple polymers are involved, one commonly employs periodic boundary conditions in terms of the minimal-image convention, which has to be considered in the above definitions for the calculation of vector differences.

In many cases, it is helpful to consider in addition a microcanonical analysis [60,61,62,63]. For Monte Carlo studies, one usually defines the conformational entropy $S\left(E_{p}\right)=k_{B}\ln\Omega \left(E_{p}\right)$ and its successive derivatives, the conformational microcanonical inverse temperature $k_{B}\beta \left(E_{p}\right)=dS\left(E_{p}\right)/dE_{p}$, or $\beta \left(E_{p}\right)=d\ln\Omega \left(E_{p}\right)/dE_{p}$ and $\gamma \left(E_{p}\right)=d\beta \left(E_{p}\right)/dE_{p}$. This encodes all relevant transitions for which the energy is a suitable reaction coordinate and allows for a classification of the transition order for finite systems [60,63]. If $\beta (E_{p})$ shows a back-bending, which corresponds to a positive peak in $\gamma (E_{p})$, the transition may be classified as first order and is accompanied by a double-peak energy probability distribution [61]. If $\beta (E_{p})$ shows an inflection point with negative slope, i.e., a negative peak in $\gamma (E_{p})$, the transition may be classified as second order instead. This analysis may be very helpful, especially when signals of several transitions overlap in the canonical ensemble. For a recent discussion of the mapping to the full microcanonical ensemble in terms of total energy, see [64]. The microcanonical analysis is particularly suitable for flat-histogram methods, because they directly yield an estimate of the density of states.

## 4. Phase Behavior of Isolated Semiflexible Polymers

The structural motifs of a semiflexible polymer depend strongly on the variation of external parameters, such as temperature or salt concentration, as well as the formulation of relevant interactions, such as excluded volume effects, hydrophobicity, etc. It is a major challenge to understand and predict the outcome of these variations, especially in the context of biopolymers or proteins. For example, the formulation of a tube-like polymer model [65,66] allowed studying the structural motifs and metastable states that arise due to the interplay of excluded volume effects, hydrophobicity and hydrogen bonding [67]. Here, thickness (defined, e.g., in terms of the global radius of curvature [68]) plays a crucial role in the formation of secondary structures, such as helices [69,70,71]. Still on this level, well-parameterized lattice protein models allow one to investigate the effect of hydrophobicity and its relation to cold denaturation [72].

Currently, there is again a growing interest in the role of knots in single polymers [73,74,75,76] and proteins [77,78,79,80,81,82,83]. This is particularly interesting in the context of DNA packing [84,85] with possible implications for DNA sequencing, especially since DNA can be interpreted as a semiflexible homopolymer [86,87,88]. Here, self-avoidance is a crucial aspect for the formation of knots by geometric hindrance, where thickness can be understood as the diameter of the coarse-grained beads. The usual picture is that knots occur with a certain probability within structural regimes, e.g., within extended coils, globules or densely-packed toroids. However, most approaches model polymers with purely repulsive monomer-monomer interactions, i.e., similar to a self-avoiding walk, or assume them to be flexible. We will show below that for specific parameter combinations, including both attractive monomer-monomer interactions and bending stiffness, knots can fully characterize stable structural regimes on their own.

In the following, we focus on the effect of bending stiffness, where the energy and length scales of the self- and mutual-attraction are fixed, but the strength of the bending penalty is varied. This includes mean-field studies [89], numerical studies of lattice models [90,91,92] and off-lattice models [93,94,95,96,97,98], analytical approaches [99] and experimental studies [100]. It was shown that the variation of a single parameter, such as the stiffness, may strongly influence the arising structural motifs and, moreover, may affect the order of the accompanying transitions. Interestingly, already, the variation of the short-range attraction range [101,102,103] may alter the structural transition of a flexible polymer to directly fold into its frozen state or even foster new low-temperature states. Similarly, a modification of the bond-interaction range alters the second-order collapse transition into a first-order condensation transition of coupled monomers [104]. Extending the model by torsional angles and adjusting a proper combination of confined bending and torsional angles again leads to the stabilization of helical structures [105,106].

### 4.1. Structural Phase Diagram

The variation of polymer stiffness inevitably leads to a modification of the structural motifs for finite temperatures. For infinite, or sufficiently high, temperatures, the polymers will behave as random coils because conformational entropy dominates the energy reduction by contact formation or stiffening. Notice that our discussion here is not in terms of the persistence length, which in the worm-like chain limit may be related to the local stiffness as $l_{p}/\sigma \approx \kappa /k_{B}T$ and connects the energy and temperature scale. This concept is not trivially applied to multiple length and energy scales, especially for self- and mutual-attraction (for a recent debate on a proper definition, see, e.g., [107]).

**Figure 1:**
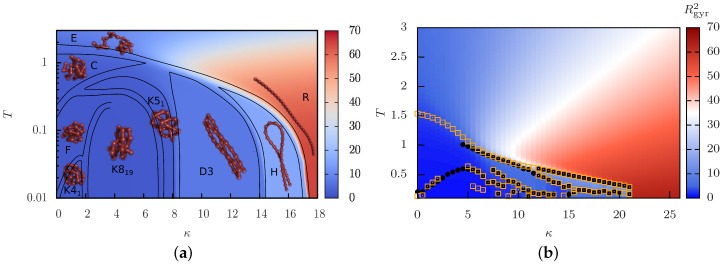
Comparison of structural phase diagrams for a single semiflexible polymer with self-attraction: (**a**) bead-stick polymer (N=28, logarithmic temperature scale, adapted from [98]); and (**b**) bead-spring polymer (N=40, linear temperature scale from parallel multicanonical simulations). Black circles and orange squares correspond to peak positions of $C_{V}$ and $dR_{\mathrm{gyr}}^{2}/dT$, respectively. The background color encodes the squared radius of gyration, i.e., the polymer extension. Representative conformations are shown for the bead-stick polymer in the respective regimes and are similar in both cases, except for the knotted regimes (bead-stick) and toroidal regimes (bead-spring [97]). The collapse transition line is here denoted as all initial temperature-driven transitions from an extended structure into a compact structure.

Basic statistical mechanics implies that lowering the temperature boosts the role of energetic minimization. The self-attractive semiflexible polymer achieves this by forming additional non-neighboring monomer contacts (local collapse) or by aligning neighboring bonds (local stiffening). Fixing the energy scale to the monomer-monomer interaction, i.e., keeping ϵ=const. in Equation (1), and varying the stiffness *κ* thus leads to a competition between local collapse and local stiffening. As an illustration, we present in Figure 1 exemplary structural phase diagrams for a bead-stick polymer (Figure 1a), adapted from [98], and a bead-spring polymer (Figure 1b), simulated with parallel multicanonical simulations. (Our parallel multicanonical simulations for κ∈[0,26] with Δκ=0.5 employ 64 cores in energy ranges that cover T∈[0.1,5]. In the final production run, we record a total of 2.56 million measurement points.) Due to our parameterizations, both models describe polymers of linear equilibrium extension $Nr_{0}=28$. The basic shape for these off-lattice polymers is quite similar and in accordance with other results [97]. Of course, the explicit temperature and stiffness at which a transition occurs is highly model-dependent. We denote the collapse transition line as all initial temperature-induced transitions from an extended (*E*) or rod-like regime (*R*) into a compact (*C*), bent (*D*) or hairpin (*H*) regime.

Details of the models become relevant again for the low-temperature conformations. For off-lattice models, this includes the formation of stable knots [98] (see Section 4.3), toroidal loops [97,99] and the arrangement of the compact frozen states, which may form icosahedral structures [97,108]. This appears to depend on many details, such as the explicit relations between the bonded and non-bonded length and energy scales, with many open questions remaining.

The structural phase diagram observed for off-lattice polymers is quite similar to the ones observed for discrete lattice polymers [89,91,92]. However, while for off-lattice polymers the collapse transition line into globular or folded structures vanishes for large stiffness, one may observe for relatively stiff lattice polymers a freezing transition temperature that further increases with stiffness. We suspect that this “freezing” transition is in fact a transition into a linear rod and should be rather denoted stiffening transition instead of folding. This would coincide with the crossover in the off-lattice model from extended (*E*) to rod-like (*R*) structures, which can be seen in Figure 1 as a color gradient from blue to red.

### 4.2. Order of the Collapse Transition Line

The influence of stiffness on the collapse transition, more explicitly the morphological variation or the type, has been a long-standing subject of investigation [89,90,91,92,93,94,95,96,97,98,99,100]. The collapse transition of a flexible polymer is a second-order phase transition in the limit N→∞ [2]. This means that the transition causes a continuous change of an order parameter. The situation becomes less clear for non-zero stiffness *κ* because the formulation of the thermodynamic limit is not obvious. Still, a common conclusion is that stiffness changes the continuous collapse transition to a discontinuous collapse or folding transition.

**Figure 2:**
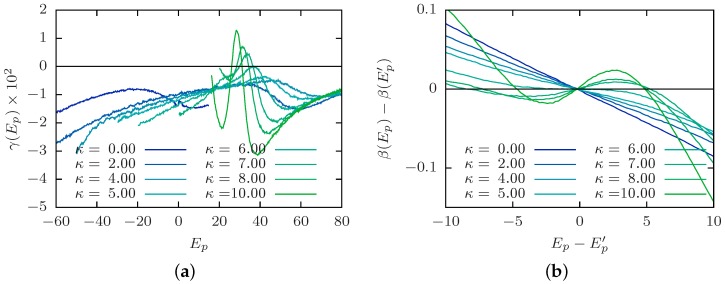
Microcanonical analysis of the collapse transition line for a bead-spring polymer (N=40). (**a**) The transition energies $E_{p}^{\prime }$ are identified as the peak location of $\gamma (E_{p})$. It can be seen that for low *κ*, the transition peak is below zero (second order), but with increasing *κ*, there is a crossover to peaks above zero (first order). (**b**) A rescaled plot of the microcanonical inverse temperature $\beta \left(E_{p}\right)-\beta \left(E_{p}^{\prime }\right)$ around the transition energy $E_{p}-E_{p}^{\prime }$ shows the crossover from second-order (no back-bending) to first-order transition (back-bending).

We mentioned above that identifying the collapse transition in the canonical analysis may be quite difficult, especially for low stiffness; there is merely a shoulder in the specific heat [109]. This introduces difficulties in the identification of the transition order, where an often employed distinction is the scaling of the specific-heat peak. The microcanonical analysis (see Section 3.4), on the other hand, provides an illustrative approach to study the order of the collapse transition. We consider the bead-spring polymer (N=40) discussed in Figure 1b of the previous subsection. Figure 2a shows the second derivative of the microcanonical entropy $\gamma (E_{p})$ for selected stiffness *κ*. As expected, we find a negative peak for flexible polymers and semiflexible polymers with low *κ*. This is a finite-size signature of a second-order transition. With increasing *κ*, the peak location shifts to higher energies, and the peak height approaches zero. There occurs a crossover at κ≈6 where the peak location starts shifting to lower energies again, and the peak height becomes positive for stiffer polymers. This corresponds to a finite-size signature of a first-order transition. In the formal definition of a phase transition, one would need to consider the limit N→∞ for which the peak height approaches zero, either from above (first order) or below (second order). However, as we mentioned before, this requires a thorough protocol as to what to fix in this limit and is an interesting future study.

Identifying the peak locations in Figure 2a as $E_{p}^{\prime }$, we show in Figure 2b a rescaled inverse microcanonical temperature $\beta \left(E_{p}\right)-\beta \left(E_{p}^{\prime }\right)$ shifted to its inflection point location. This serves as a good illustration of the crossover from a continuous second-order collapse transition for rather flexible polymers (low *κ*) to a discontinuous first-order collapse (or folding) transition for stiffer polymers (high *κ*) reflected by the prominent back-bending. The crossover value κ≈6 is consistent with the change of structural motifs in polymer aggregation [110]. Moreover, the change in transition order may be related to similar observations for varying interaction length scales [101,102,103], if one considers that the stiffness induces an effective linear length scale along the polymer, which competes with the monomer-monomer interaction length scale. It is a worthwhile future study to investigate how this crossover value depends on polymer length, number and model, connected to the involved length and energy scales.

### 4.3. Knots as Stable Phase

The most surprising regions of the phase diagram for the semiflexible bead-stick polymer in Figure 1a are stable polymer knots, labeled by “K”. These structural phases are novel in that the emerging knots are thermodynamically stable and may hence be considered as a topological order parameter [98]. Their properties are considerably different from those of knots frequently observed in the swollen and globular phases of flexible polymers [73,74,75,76], where they form just by chance and disappear again after a while.

**Figure 3:**
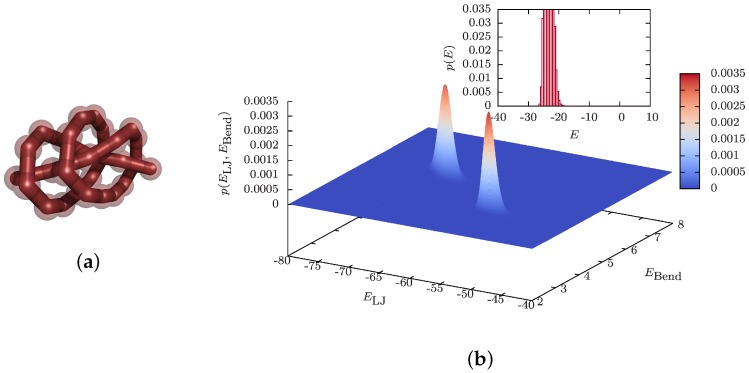
Characterization of the transition into polymer knots for a semiflexible bead-stick polymer (N=28) adapted from [98]. (**a**) Typical knot of type $5_{1}$ (at κ=7.50, T=0.045). (**b**) Two-dimensional (potential) energy histogram $p(E_{\mathrm{LJ}},E_{\mathrm{Bend}})$ at the D3–K5${}_{1}$ transition for κ=8.0 at T=0.18, signaling clear phase coexistence. The inset shows the one-dimensional histogram p(E) of the total (potential) energy $E=E_{\mathrm{LJ}}+\kappa E_{\mathrm{Bend}}$, which corresponds to a projection along the diagonal of the two-dimensional histogram. In this projection, the two peaks fall on top of each other, so only a single peak is visible in p(E).

Closer inspection reveals that the knotted conformations in the phase diagram for the 28-mer can be identified according to the usual classification scheme as $C_{n}=4_{1}$, $5_{1}$ and $8_{19}$. Here, the integer *C* counts the minimal number of crossings of any projection of a knot onto a two-dimensional plane, and the subscript *n* distinguishes topologically-different knots characterized by the same integer value *C*. A typical example found in the simulations, the $5_{1}$ knot, is shown in Figure 3a. These are so-called torus knots, which are known to form preferentially in viral DNA [86]. For the identification of the knot type, one determines for each polymer conformation the Alexander polynomial Δ(t). For the definition and properties of the Alexander polynomial and a detailed description of mathematical knot theory in general, see the book by Kauffman [111]. A useful variant is described in [112], in which a specific product $\Delta_{p}\left(t\right)\equiv \left|\Delta \left(t\right)\times \Delta \left(1/t\right)\right|$ of the Alexander polynomial Δ(t) is evaluated at t=−1.1. This proved to identify the smaller knots uniquely. Of course, in a strict mathematical sense, the identification of knots in an open polymer is topologically not well defined. To circumvent this problem one first has to apply a suitable (virtual) closure prescription. For a detailed discussion, see [98,112].

A related observation concerns the nature of the transitions between the knot phases and the frozen or bent phases. Since these transitions connect two structured states, one would expect first-order-like characteristics, similar to other solid-solid transitions. However, as the inset of Figure 3b shows for the D3–K5${}_{1}$ transition, this expectation may not be true since the (potential) energy distribution p(E) exhibits only a single peak, suggesting a second-order-like transition. There is no indication for the typical double-peak structure at a first-order-like phase transition and, hence, no signal of latent heat [113,114]. The true nature of the transition is only revealed when one considers the two-dimensional (potential) energy distribution $p(E_{\mathrm{LJ}},E_{\mathrm{Bend}})$, for which indeed, two clearly separated peaks are visible in Figure 3b [98]. The peak in front corresponds to the (unknotted) bent phase D3 and the other in the back to the phase characterized by the K5${}_{1}$ knot, presented in Figure 3a. The total (potential) energy $E=E_{\mathrm{LJ}}+\kappa E_{\mathrm{Bend}}$ is the projection along the diagonal of this two-dimensional histogram along which the two peaks fall on top of each other, which explains why only a single peak shows up in p(E) and no latent heat is observable.

Previous studies of semiflexible polymers have reported no knot phases, considering a particular bead-spring model [97]. Our own test simulations of the bead-spring model Equations (1)–(3) lead to the same conclusion; see Figure 1b. We suspect that the reason for this difference lies in the choice of the ratio of length scales, i.e., the equilibrium length of neighboring bonds $r_{b}$ and of non-neighboring monomer-monomer interactions $r_{n}$ (determined from the minimum of the Lennard–Jones potential in Equation (1)). This ratio $r_{n}/r_{b}$ is typically set to unity (see Figure 1b and [97]), where bent conformations are energetically favored over knots. For our bead-stick parameterization, however, $r_{n}/r_{b}\approx 1.12$, which seems to induce the stable knots. To verify this conjecture, more work is necessary.

## 5. Aggregation of Dilute Semiflexible Polymers

Aggregation in dilute solutions of semiflexible polymers describes the competition between an entropic soluble regime where polymers are effectively isolated and an energetic aggregated regime where polymers form clusters or aggregates. Here, many questions are still open, and one often considers explicit heteropolymers/peptides or proteins connected to specific problems. In this context, there has been much recent effort, including full atomistic approaches [115], heteropolymer models [62,116,117,118], tube-like homopolymers [119], extended lattice models [120] and even single-site lattice models [121,122,123], to name only a few. Of course, there is a huge amount of molecular dynamics studies. In the following, we focus on the statistical mechanics of aggregation in dilute homopolymer models. Flexible polymers were shown to exhibit nucleation hierarchies [124] known from droplet formation. An interesting future question is how this analogy carries over to larger system sizes or to the underlying process itself [123,125,126].

Extending the discussion to multiple chains calls for the necessity to formally introduce a non-zero density ρ=NM/V, with *M* the number of polymers of length *N* and *V* the system volume. The system may be commonly confined in a cubic periodic box of linear length *L* with $V=L^{3}$ or in a geometric confinement, e.g., in a sphere of radius $R_{S}$ with $V=\frac{4\pi }{3}R_{S}^{3}$. We consider only dilute homopolymers, such that both cases are in fact equivalent in the limit of large system sizes because of the translational invariance in the former and the merely effective repulsive interactions in the latter. Still, the introduction of translational entropy increases the computational effort extremely and results in a competition of entropy maximization by spreading of polymers with energy minimization by forming an aggregate. In the simulation protocol and the analysis setup of our own polymer studies, we tried to connect as closely as possible to recent related investigations of particle condensation [127]. Here, generalized-ensemble methods may be particularly helpful. However, it is important to include update moves, such as long-range polymer displacements, to allow a decent sampling of the void space.

### 5.1. End-to-End Order Parameter

**Figure 4:**
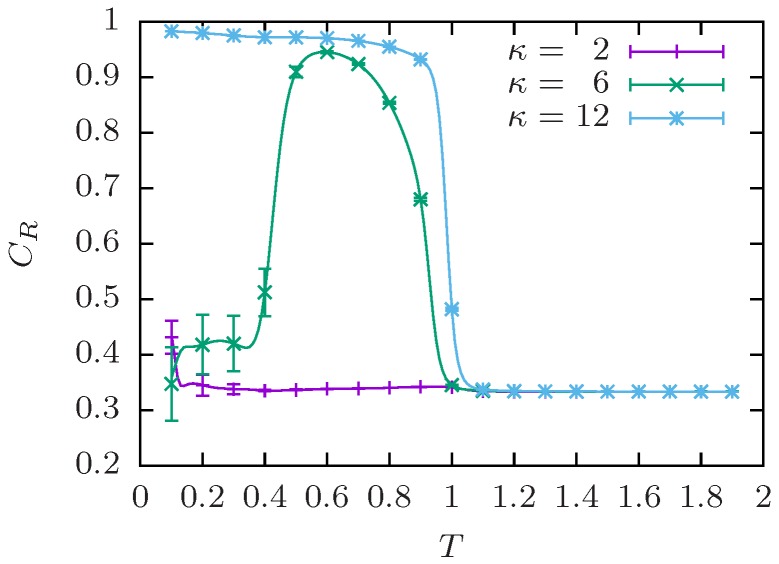
End-to-end correlation parameter $C_{R}$ for M=4 semiflexible polymers of length N=13 at the selected bending stiffness values. The aggregate morphology changes from uncorrelated (κ=2) over initially correlated (κ=6) to correlated (κ=12). For a full *κ* overview with exemplary conformations, see Figure 5.

For the aggregation of semiflexible polymers, it is useful to introduce an end-to-end correlation parameter $C_{R}$ measuring the correlation between the end-to-end vectors ${\hat{R}}_{i}$ (normalized to unit length). This is similar to a nematic order parameter and allows one to distinguish between amorphous aggregates, where ${\hat{R}}_{i}$ are uncorrelated, and bundles, where ${\hat{R}}_{i}$ are aligned. We choose:

(8) $$C_{R}=\frac{2}{M(M-1)}\sum_{i<j}{\left({\hat{R}}_{i}\cdot {\hat{R}}_{j}\right)}^{2},$$

such that for completely aligned bundles $C_{R}=1$ and for uncorrelated polymers $C_{R}=1/3$, e.g., polymers in the fragmented regime or in amorphous aggregates [110]. An illustration for M=4 polymers of length N=13 is shown in Figure 4. With increasing polymer number, the probability of complete alignment may decrease either due to twisting of bundles (see the discussion below) or by less ordered bulk aggregates. However, for the system sizes discussed in this context, $C_{R}$ serves as a good distinction between amorphous aggregates and polymer bundles.

### 5.2. Structural Motifs Induced by Semiflexibility

**Figure 5:**
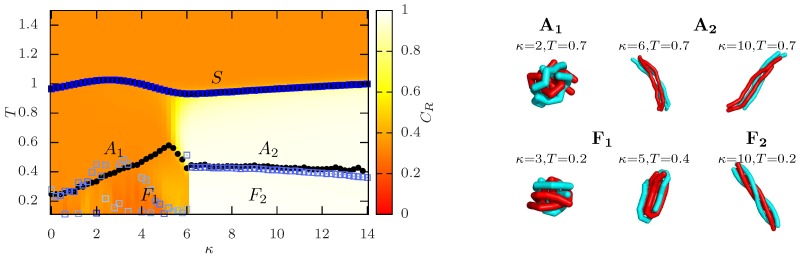
Structural phase diagram of M=4 polymers of length N=13 in the temperature-stiffness plane. The color map encodes the end-to-end correlation parameter from uncorrelated ($C_{R}=1/3$, orange) to correlated ($C_{R}=1$, white). Black dots denote peaks in the specific heat; blue squares indicate peaks in the phase-separation parameter with signal strength encoded in the color intensity. From the solute (S) regime, the polymers aggregate into amorphous aggregates ($A_{1}$) or polymer bundles ($A_{2}$) for low or high bending stiffness, respectively. At even lower temperatures, more ordered structures occur ($F_{i}$). Representative conformations are shown next to the diagram.

In Section 4.1, we have seen that stiffness results in the variation of structural motifs for isolated polymers, ranging from spherically-symmetric globular conformations for flexible polymers to hairpin or multiple-bent conformations for stiffer polymers. Here, we want to discuss the effect of semiflexibility on the structural motifs of aggregates, including M=4 bead-spring polymers of length N=13 in a cubic box with density $\rho =10^{-3}$ and minimal-image convention (the system setup and model parameters are completely analogous to [110]). This system is small and dilute enough that all polymers will be included in the aggregate after the temperature-driven transition. We will qualitatively recapture and, thus, extend the results for M=2 and M=8 polymers from [110]. Figure 5 shows the structural phase diagram for the considered system. The background color encodes the end-to-end correlation parameter $C_{R}$, and the black dots, as well as blue squares denote possible transition points from peaks in the specific heat $C_{V}$ or the phase-separation parameter $\Gamma^{2}$ [116,124], respectively. As expected, we observe for high temperatures a solute or fragmented regime, where the polymer may be considered isolated, and the structural properties follow the single-chain behavior. Upon a temperature decrease, the system shows a stiffness-dependent response in the formation of aggregates. Flexible polymers (κ<6) are seemingly uncorrelated within the aggregate, which may be called amorphous. A further temperature decrease may enforce coiled structures, but the polymers remain uncorrelated. On the other hand, stiffer polymers (κ>6) form bundles, which is reflected in the end-to-end correlation. Lowering the temperature even further results in twisted bundles known from biopolymer systems, e.g., from amyloid protofibrils [128] or actin networks [129]. This is consistent if we interpret the bead-spring model as a coarse-grained model of either protofibrils or actin filaments. Moreover, a clear understanding of bundling is of importance for the design of specific polymeric materials [130]. In this limit of rather stiff polymers, the structural properties may be well-approximated by worm-like chain-based approaches, e.g., for the study of unbinding transitions [131,132] and the twisting of filaments [133,134,135].

**Figure 6:**
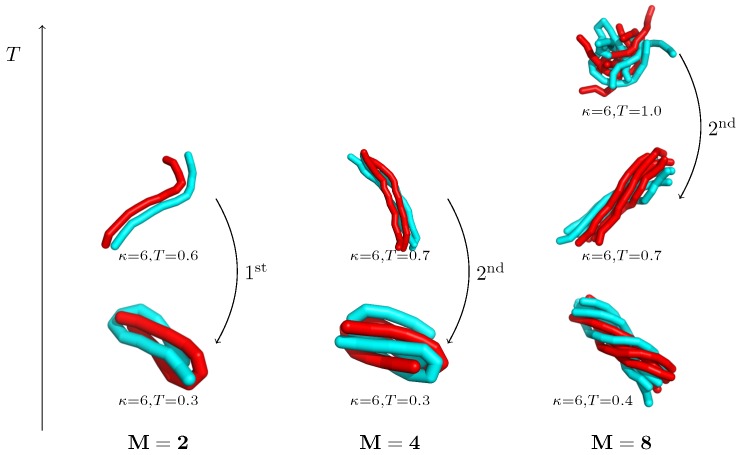
Size-dependence of the sub-aggregation re-ordering transitions within the aggregate for κ=6. Representative conformations are presented in the intermediate-stiffness regime for M={2,4,8} polymers of length N=13. M=2 polymers show a first-order-like bundle-to-hairpin structural transition [110], while the finite-size transition for M=4 polymers from elongated bundles to bundled hairpins is second-order like. M=8 polymers show a second-order-like amorphous-to-bundle transition followed by the formation of twisted bundles at even lower temperatures [110].

There exists an intermediate regime (κ≈6), where the aggregate re-orders between correlated and uncorrelated structural motifs in sub-aggregation transitions, i.e., below the initial aggregation transition. The resulting structures and the order of the sub-aggregation transition therein strongly depend on the number of involved chains [110]. An illustration is provided in Figure 6 for M={2,4,8} polymers at temperatures below the aggregation transition. For two polymers, the sub-aggregation transition from “bundles” into entangled hairpins is first-order like, while already for four polymers, the transition into bundled hairpins shows second-order-like signatures. The scenario changes for eight polymers, which start by first forming amorphous aggregates in the intermediate regime before rearranging into bundles in a second-order-like transition. The latter behavior may be expected to be relevant for increasing polymer numbers.

The primary aggregation transition is of first order over the full stiffness range due to the competition between entropy maximization and energy minimization. This is connected to a free-energy barrier of aggregation, which was shown to gradually increase with stiffness [110]. Thus, the formation of polymer bundles requires overcoming a higher barrier than the formation of amorphous aggregates. This is consistent with observations of increasing lag times in the formation of amyloid fibrils compared to amorphous aggregates [136].

### 5.3. Competition between Single-Chain Collapse and Many-Chain Aggregation

If dilute semiflexible polymers at high temperature behave as isolated chains, then they inevitably undergo the collapse transition discussed in Section 4.1. An immediate question arises: How does the discontinuous aggregation transition interfere with this single-chain transition, which as we have seen varies from a continuous to a discontinuous transition with stiffness.

Using a dilute setup, it was observed that collapse and aggregation are not separate processes anymore [124], similar as for protein folding and binding [116]. It was argued that the structural motif of the aggregate governs the collapse or folding behavior of the individual polymers. Similar results were found for specific lattice proteins, where the single-chain folding transition was below the binding transition [137]. However, the aggregation or binding transition depends on the density [125,137] as $\beta_{\mathrm{agg}}=a_{1}\ln\rho +a_{2}$, which follows from entropic (ideal-gas based) arguments. It was argued that this may indeed lead to a folding-docking mechanism [137]. On the other hand, recent results for semiflexible polymers indicate that the isolated chains follow the collapse transition up to the point of aggregation, where the collapse is reversed in order to form energetically-favorable aggregates [125,126]. This is consistent with the self-templated nucleation observed for the aggregation of proteins and peptides [119]. In general, the dominance of global versus local structure strongly depends on the relation of intra- and inter-chain interactions. If the energy reduction from aggregation is more beneficial than from attached globules, then multi-chain aggregation may be expected to reverse single-chain collapse. These considerations are for systems in equilibrium. If such a rearrangement can actually be observed in experiments, however, depends on the involved free-energy barriers and corresponding transition time scales.

**Figure 7:**
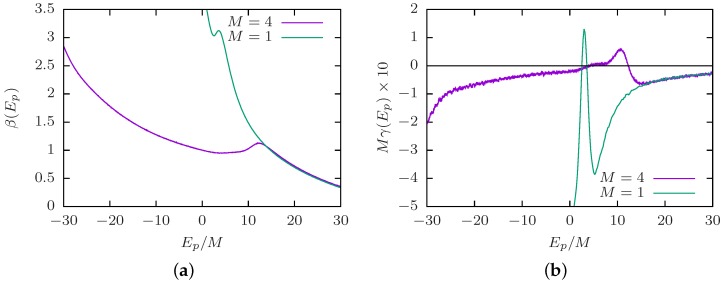
Microcanonical illustration of the competition between single-polymer collapse and many-polymer aggregation on the example of *M* semiflexible bead-spring polymers (κ=4, N=13) showing (**a**) the microcanonical inverse temperature $\beta (E_{p})$ and (**b**) its derivative $\gamma (E_{p})$. The stiffness was chosen to be stiff enough to yield a first-order-like collapse transition; compare also Figure 1 and Figure 5. Thus, both collapse and aggregation show a back-bending in $\beta (E_{p})$ and a positive peak in $\gamma (E_{p})$.

To illustrate this behavior, Figure 7 shows a microcanonical analysis of M=4 semiflexible bead-spring polymers of length N=13 at density $\rho =10^{-3}$ (as in Figure 5) compared to a single chain (the data for the single chain are again obtained from parallel multicanonical simulations as in Section 4.2). The stiffness was chosen to be stiff enough to yield a first-order-like collapse transition with a back-bending in $\beta (E_{p})$ and a positive peak in $\gamma (E_{p})$; compare Section 4.2. As observed in the canonical analysis of [125,126], the high-temperature or high-energy regime of a many-chain system coincides with the single-chain behavior or isolated chain regime. Lowering the temperature or energy, both curves coincide until the point of aggregation, a first-order finite-size transition with back-bending in $\beta (E_{p})$ and positive peak in $\gamma (E_{p})$. As for the continuous collapse transition in flexible polymers, this completely dominates the single-chain collapse or folding transition. As can be seen on the average-energy scale (*x*-axis), the aggregation leads to much smaller energies. It may thus be expected for the chosen parameters that many-chain aggregation dominates the single-chain behavior even for very low densities, for which the aggregation branch in the microcanonical inverse temperature should merely shift to higher *β*. In the present case, the energy scales of inter- and intra-chain interactions are identical, and a systematic study of the inter- and intra-chain interactions may shed some light on the effect of this choice.

## 6. Conclusions

We provided a brief overview about the structural transitions and motifs that occur in a statistical mechanics description of dilute semiflexible polymers with self- and mutual attraction in a solvent. The solvent quality is here directly linked to the temperature scale. Dilute polymers undergo a continuous collapse transition if the stiffness is low, which changes to a discontinuous transition into folded conformations for increasing stiffness. We illustrated this crossover by means of microcanonical analyses. The further increase of stiffness causes merely a low-temperature stiffening of the single chain.

Interestingly, a mismatch of interaction length scales seems to induce a novel structural phase of thermodynamically-stable knots of various types. Their properties are considerably different from those of knots observed in the swollen and globular phases of flexible polymers, which form by chance. Intriguingly, the transitions into these knotted conformations from other structured states happen with almost no latent heat, although we observed a clear phase coexistence. This suggests that the knot type may be considered as a topological order parameter.

With increasing density, multiple dilute semiflexible polymers start to form aggregates at low temperature or in bad solvent. We demonstrated that an end-to-end correlation order parameter is suitable to identify the resulting structural motifs. Rather flexible polymers form amorphous, uncorrelated structures while for increasing stiffness polymer bundles are forming. With decreasing temperature, these become twisted for reasons of energy minimization, a motif known from biological systems. The primary aggregation transition is a first-order-like transition, accompanied by a free-energy barrier that increases with stiffness. Since the individual polymers above the aggregation behave as isolated chains, they also undergo a collapse transition upon temperature decrease. The resulting competition of energy scales is dominated by the process of aggregation if the inter-chain interactions are reasonably strong. In this case, aggregation results in a partial reversion of the collapse process in order to form the equilibrium aggregate motif. Of course, the intra-polymer interaction may be tuned in such way that pre-folded chains attach to an aggregate, known from polymer and protein crystallization.

We have shown that many questions about the structural phases of semiflexible polymers have been answered using Monte Carlo methods and proper analysis techniques. However, many other questions remain still open or have only been raised by recent developments. In the future treatment of these problems, generalized-ensemble Monte Carlo simulations seem to be a reliable partner.
